# Auranofin coated catheters inhibit bacterial and fungal biofilms in a murine subcutaneous model

**DOI:** 10.3389/fcimb.2023.1135942

**Published:** 2023-05-29

**Authors:** LewisOscar Felix, Cutler Whitely, Nagendran Tharmalingam, Biswajit Mishra, Noel Vera-Gonzalez, Eleftherios Mylonakis, Anita Shukla, Beth Burgwyn Fuchs

**Affiliations:** ^1^ Division of Infectious Diseases, Rhode Island Hospital, The Miriam Hospital, Alpert Medical School and Brown University, Providence, RI, United States; ^2^ Center for Biomedical Engineering, School of Engineering, Institute for Molecular and Nanoscale Innovation, Brown University, Providence, RI, United States

**Keywords:** antimicrobial catheter, auranofin, biofilm, *Candida albicans*, drug-coated catheter, *Staphylococcus aureus*

## Abstract

Microbe entry through catheter ports can lead to biofilm accumulation and complications from catheter-related bloodstream infection and ultimately require antimicrobial treatment and catheter replacement. Although strides have been made with microbial prevention by applying standardized antiseptic techniques during catheter implantation, both bacterial and fungal microbes can present health risks to already sick individuals. To reduce microbial adhesion, murine and human catheters were coated with polyurethane and auranofin using a dip coating method and compared to non-coated materials. Upon passage of fluid through the coated material *in vitro*, flow dynamics were not impacted. The unique antimicrobial properties of the coating material auranofin has shown inhibitory activity against bacteria such as *Staphylococcus aureus* and fungi such as *Candida albicans*. Auranofin coating on catheters at 10mg/mL reduced *C*. *albicans* accumulation *in vitro* from 2.0 x 10^8^ to 7.8 x 10^5^ CFU for mouse catheters and from 1.6 x 10^7^ to 2.8 x 10^6^ for human catheters, showing an impact to mature biofilms. Assessment of a dual microbe biofilm on auranofin-coated catheters resulted in a 2-log reduction in *S*. *aureus* and a 3-log reduction in *C*. *albicans* compared to uncoated catheters. *In vivo* assessment in a murine subcutaneous model demonstrated that catheters coated with 10 mg/mL auranofin reduced independent *S*. *aureus* and *C*. *albicans* accumulation by 4-log and 1-log, respectively, compared to non-coated catheters. In conclusion, the auranofin-coated catheters demonstrate proficiency at inhibiting multiple pathogens by decreasing *S*. *aureus* and *C*. *albicans* biofilm accumulation.

## Introduction

Medical devices provide significant advances to improve human health but present an opportunity for infection by creating a portal for microbes to enter the patient’s bloodstream and potentially compromise recovery and health (Feneley et al., 2015). In the United States, about 150 million intravascular catheters are implanted yearly to support patients with renal failure, monitor hemodynamics, administer medications, and provide nutritional support (Shah et al., 2013). However, 250,000 catheter-related bloodstream infections (CRBIs) have been associated with intravascular catheters (Marik et al., 2012; Mccoy et al., 2017). In emergency rooms and intensive care units, central venous catheter insertion is associated with 5.3 bloodstream infections per thousand days (Gahlot et al., 2014). In addition to bloodstream infections, catheters present urinary tract infection vulnerability, for which catheter-associated urinary tract infections (CAUTI) account for approximately 40% of device-related infections in hospitalized patients (Feneley et al., 2015). Among urinary tract infections acquired in the hospital, 75% are catheter-related (CDC, 2015). During antineoplastic chemotherapy infusion, catheters provide a portal to bacteria and fungi by subverting the natural skin that serves as a barrier and essential defense mechanism, creating a niche for microbe adhesion and colonization of microbes derived from the skin microbiome or the external environment (Douglas, 2003; Neugent et al., 2020).

In most cases, CRBI and CAUTI are caused by pathogens that form biofilms (Lynch and Robertson, 2008), which are the complex structure of microorganisms that contains proteins, nucleic acids, and polysaccharides. Compared to planktonic cells, biofilms are more resistant to conventional antimicrobials through their recalcitrant nature and employment of efflux pumps (Gilbert et al., 2002). As a result of biofilm formation, bacteria (e.g., *Staphylococcus aureus*, *Staphylococcus epidermidis*, and *Pseudomonas aeruginosa*) and fungi (e.g., *Candida albicans*) can cause infections, such as bloodstream infections that are challenging to treat (Chandra et al., 2001; Donlan, 2001; Mah and O’toole, 2001; Yousif et al., 2015; Brindhadevi et al., 2020; Wolcott, 2021).

Among catheter-related microbial infections, *S. aureus* and *C. albicans* are the most common catheter-dwelling, biofilm-forming bacterial and fungal pathogens, respectively (Esposito et al., 2013; Laverty et al., 2015; Kohse et al., 2019). Strenuous efforts have been made to reduce the number of catheter-related infections by employing stringent local sterilization and catheter implant procedures (O’grady et al., 2002; Panepinto et al., 2021; Selby et al., 2021) however, infections still occur. Central venous catheters are risk factors for acquiring *C*. *albicans* catheter-related bloodstream infections (CRBI), especially among immunocompromised patients. Catheter-related fungemia can be associated with parenteral nutrition, central lines, and other implanted catheters. Infection rates can vary between the global region and even hospitals. Overall, *C*. *albicans* as well as other *Candida* species, pose a risk of infection. Despite best practices, the skin microbiome often serves as the inoculum reservoir. In the UK, CRBI accounts for 10 to 20% of hospital-acquired infections (Gahlot et al., 2014). A review by Gahlot and colleagues finds *Candida* species as the infecting agents in 11.7 to 16% of central venous catheters (Gahlot et al., 2014). In a single-site study from a pediatric intensive care unit in Turkey, *C*. *albicans* accounted for 16% of the central line *Candida* infections (236 in total) over a 11-year span (2009-2019) (Devrim et al., 2022).

Surface modification and/or coating catheters with bioactive molecules (peptides, small antimicrobial molecules) provide means to prevent the colonization of *S. aureus* and *C. albicans* under *in vitro* and *in vivo* conditions (Li et al., 2014; Francolini et al., 2017; Liu et al., 2019b; Pathak et al., 2019; Balaure and Grumezescu, 2020). Application of bioactive molecules like antimicrobial agents, small molecules, metallic nanoparticles, enzymes, or antiseptic agents helps to slow the release of the bioactive molecule causing bacterial death and preventing biofilm formation (Stenger et al., 2016; Vaterrodt et al., 2016; Lewisoscar et al., 2018; She et al., 2020; Lewisoscar et al., 2021).

In the present study, auranofin, a Food and Drug Administration (FDA)-approved antirheumatic drug that exhibits antibacterial activity against Gram-positive bacteria and some fungi, was selected to coat catheters (Fuchs et al., 2016; Liu et al., 2019a; She et al., 2020; Felix et al., 2021). Auranofin shows antibacterial activity against Gram-positive bacteria (*Bacillus subtilis*, *Enterococcus faecalis*, *Enterococcus faecium*, *Mycobacterium tuberculosis*, and *S*. *aureus*) and fungi like *C*. *albicans*, *Candida glabrata*, *Candida tropicalis*, *Candida neoformans*, *Cryptococcus gattii* (Siles et al., 2013; Fuchs et al., 2016; Torres et al., 2016; Thangamani et al., 2017; Wiederhold et al., 2017; She et al., 2020; Felix et al., 2021). Auranofin exhibits antibacterial activity by inhibiting thioredoxin reductase, which protects against reactive oxygen species (May and Yu, 2018; Liu et al., 2019b). Disruption of the bacterial thioredoxin reductase and redox balance causes bacterial cell death.

In our previous study, we coated catheters with polyurethane (PU) and auranofin and tested the efficacy and stability of the coating for antibacterial and antibiofilm activity against *S. aureus in vitro* (Liu et al., 2019b). Expanding on this previous study, we analyzed the flow dynamics of auranofin-coated catheters. Additionally, since auranofin has a unique capacity to inhibit bacterial and fungal pathogens (She et al., 2020), auranofin-coated catheter resistance potential was evaluated for both *S*. *aureus* and *C*. *albicans* using a murine subcutaneous model.

## Methods

### PU catheter coating

Using the methods previously published by (Liu et al., 2019a), catheters were coated with antimicrobial agents. In brief, mouse and human catheters were coated with auranofin (3 mg/mL and 10 mg/mL), fluconazole (10 mg/mL), and vancomycin (15 mg/mL). Negative control catheters were coated with THF + PU alone, absent any antimicrobial agents. Human (14G x 2”, Exel Safelite Catheters, 26726, Radiopaque teflon material) and mouse (14G x 2”, Surflo I.V. Catheters, SR-OX1451CA, Polyurethane material) catheters were cut into 1 cm segments using a sharp scalpel and submerged in a 20 mL scintillation vial containing 3 mL of THF + PU + auranofin/fluconazole/vancomycin. The setup was placed on a shaker overnight at room temperature. Post incubation, catheters were air-dried in a fume hood overnight and then stored at 4°C until further use.

### Fluid flow dynamics with antimicrobial coated catheters

The fluid flow rate of auranofin-coated and uncoated human and mouse catheters was evaluated using a syringe pump (Harvard Apparatus, Holliston, MA). Assessment entailed providing a constant flow of liquid (phosphate buffered saline (PBS)) and measuring the capture volume.

For both human and mouse catheters, assessment groups included: untreated catheters, THF-coated catheters, PU dissolved in THF catheters, catheters coated with auranofin (3 and 10 mg) suspended in THF + PU coating, vancomycin (15 mg) suspended in THF + PU coating, and fluconazole (10 mg) suspended in THF + PU coating. The flow of PBS through human and mouse catheters was determined by keeping the volume of liquid constant. A 20 mL syringe was filled with 20 mL (the syringe was filled 3 times) of PBS and connected to a catheter adapter that married the catheters to the syringe tip, connecting the devices to the syringe pump **(** **Supplemental figure 1** **)**, which was then set to run at a flow rate of 1 mL/min for 10 min at a 50% force level to assess the final volume captured during a fixed period.

By contrast, to quantify the time needed to collect a fixed volume of liquid, a syringe was filled with PBS, locked into the syringe pump, and run for 10 minutes. The flow rate was set at 1 mL/min with 50% force. The volume of PBS collected was measured *via* an analytical scale and confirmed by measuring volume in a graduated tube.

### Murine subcutaneous implant model to measure *Staphylococcus aureus* biofilm accumulation

Female BALB/c mice (n=5/group) were used according to the protocol approved by the Institutional Animal Care and Use Committee. The 1 cm drug-coated catheters were put inside culture tubes containing luminescent *S. aureus* USA300 (Dai et al., 2013) logarithmic culture and incubated for 2 h with agitation. To implant, the catheters, BALB/c female mice (Charles River, Wilmington, MA.) were anesthetized *via* intraperitoneal (IP) injection with ketamine (100 mg/kg) and xylazine (10 mg/kg). Mouse flanks were shaved, and the skin was sterilized topically using betadine and isopropyl alcohol. A 5 mm skin incision was made using sterile scissors to create a subcutaneous tunnel for the 1 cm catheter previously seeded with bacteria and the incision site was then closed with surgical staples. Two procedures were performed per animal with placement of two catheters per animal, one on each flank.

Bacterial colonization was analyzed using the live animal imaging system (IVIS Lumina, Series III; Perkin Elmer, Waltham, MA, USA) at various time points following inoculation: 24, 48, and 72 h post insertion. Total photon emission from defined regions of interest within the images of each mouse was quantified using Living Image software (Perkin Elmer, Waltham, MA). After the final imaging time, mice were sacrificed, the catheters were surgically removed, sonicated in 1 mL tryptic soy broth (TSB), and the bacterial load was enumerated after serial dilution and plating on TSB agar plates. Bacteria burden from the experimental catheters was compared using One-way ANOVA using GraphPad Prism Version 6.04 (GraphPad Software, La Jolla, CA).

### *Candida albicans* biofilm accumulation on coated catheters

Both mouse and human catheters were assessed for fungal biofilm formation and accumulation of microbial cells after placing uncoated and coated 1 cm segments in 12-well plates and adding 2 mL of bovine serum (BS) (ThermoFisher Scientific, Waltham, MA) that were then incubated overnight at 37 °C with agitation (150 rpm) in an orbital shaker. Post incubation, the BS was removed through aspiration, and the catheters were washed with 2 mL of PBS. Catheters were then transferred to a fresh plate with 2 mL of spider medium (10 g of Difco nutrient broth; 10 g of Mannitol; 4g of K_2_HPO_4_; pH to 7.2; autoclave in 1 L of water) and inoculated with 40 µL (OD_600 =_ 0.5) of an overnight culture of constitutively expressed green fluorescent protein (GFP) *C. albicans* MLR62 (Nobile and Mitchell, 2005). Seeded cultures were incubated for 90 min at 150 rpm on an orbital shaker, allowing fungal cells to adhere to catheter surfaces, then washed with PBS before being transferred to a fresh 12-well plate containing 2 mL of Spider broth. Catheters were allowed to incubate with agitation for 60 hours, allowing the formation of mature biofilms before being gently washed with 2 mL of PBS. While suspended in PBS, the catheters were incubated for 10 min in a sonicating water bath (Model: Branson 2800, SonicsOnline, Richmond, VA, USA) (with an interval of 5 sec after every 30 sec of sonication). A 100 µL aliquot for each sample was transferred to a fresh 96-well plate (Corning, ThermoFisher Scientific, Waltham, MA). The released fungal cells were quantified at 488 nm excitation and 535 nm emission using SpectraMax i3X (Molecular Devices, San Jose, CA). The remaining sonicated slurry was serially diluted in PBS and plated on a YPD agar, then incubated for 18 h before enumerating the colony forming units (CFU).

### *Staphylococcus aureus* - *Candida albicans* dual biofilm formation on catheters

Both mouse and human catheters were assessed for dual biofilms (*S*. *aureus* - C. *albicans* biofilm) by placing segments in 12-well plates and adding 2 mL of bovine serum (BS) (ThermoFisher Scientific, Waltham, MA). Catheters were incubated overnight at 37°C with agitation (150 rpm) in an orbital shaker. Post incubation, the BS was removed through aspiration, and the catheters were washed with 2 mL of PBS. The catheters were then transferred to a fresh plate 2 mL plate with 40 µL of *C. albicans* MLR62 (1 × 10^6^ cells/mL) (Nobile and Mitchell, 2005) and *S. aureus* USA 300 (1 × 10^7^ cells/mL) in 1:1 v*/*v YPD/BHI (2 mL) media. Seeded cultures were incubated for 90 min at 37 °C at 150 rpm on an orbital shaker, allowing fungal/bacteria cells to adhere to catheter surfaces, then washed with PBS before being transferred to a fresh 12-well plate containing 2 mL of Spider broth. Catheters were allowed to incubate with agitation for 60 hours, allowing the formation of mature biofilms, before being gently washed with 2 mL of PBS. While suspended in 2 mL of PBS, the catheters were sonicated for 10 min (with an interval of 5 sec after every 30 sec of sonication) in a water bath sonicator (Model: Branson 2800, SonicsOnline, Richmond, VA, USA). A 100 µL aliquot for each sample was serially diluted. Fungal cell growth was selected on a YPD plate containing vancomycin (15 mg/mL), and bacterial cells were set on TSB media containing fluconazole (25 mg/mL). The plate was incubated at 37°C for 18 h, and the CFU was enumerated.

### Murine subcutaneous catheter model infected with *Candida albicans* biofilm

Female BALB/c mice (n=5/group) were used according to the protocol approved by the Institutional Animal Care and Use Committee. Mouse catheters (uncoated, THF+PU, auranofin 3 mg + PU 50 mg, auranofin 10 mg + PU 50 mg, and fluconazole 10 mg + PU 50 mg) were treated overnight in 2 mL of FBS and inoculated with 1x10^4^ CFU/mL of *C. albicans* MLR62 and incubated at 37°C for 90 min (Nobile and Mitchell, 2005). On day 1, the BALB/c female mice (Charles River, Wilmington, MA.) were anesthetized *via* IP injection with ketamine (100 mg/kg) and xylazine (10 mg/kg). The mouse flanks were shaved, and the skin was sterilized topically using betadine and isopropyl alcohol. A 5 mm skin incision was made using sterile scissors to create subcutaneous tunnels on both animal flanks for 1 cm catheters that were previously seeded with *C. albicans* MLR62. Upon completion, the incisions were closed with steel wound clips. Control animals were implanted with two drug-free catheters. Post-procedure, the animals were revived with atipamezole (1 mg/10 mg of xylazine used in a 100 PL volume) *via* IP injection. After catheter implantation and upon animal revival, the mice were given buprenorphine SR at 0.5 to 1 mg/kg *via* SQ injection.

After the 4-day evaluation period, animals were anesthetized with ketamine-xylazine and euthanized *via* exsanguination by cardiac puncture to collect blood (~1 mL). Subsequently, the catheters were removed from the euthanized mice and the blood was plated on YPD media to enumerate *C. albicans* MLR62 CFU. The retrieved catheters were sonicated in 1 mL YPD broth, and the fungal load was enumerated after serial dilution and plating on YPD agar plates (**Supplemental figure 2** **)**.

## Results

### Fluid flow dynamics through human and mouse catheters are not altered by the drug-coating process

PBS was passed through coated catheters and analyzed using various antimicrobial coating agents to determine if the coating process impedes fluid flow. Flow dynamics were assessed for mouse and human catheters coated with auranofin (3 and 10 mg/mL), vancomycin (15 mg/mL), and fluconazole (10 mg/mL). Coated catheters were studied in three aspects, namely: time, volume, and mass.

### Fluid flow volume

**Figure 1:**
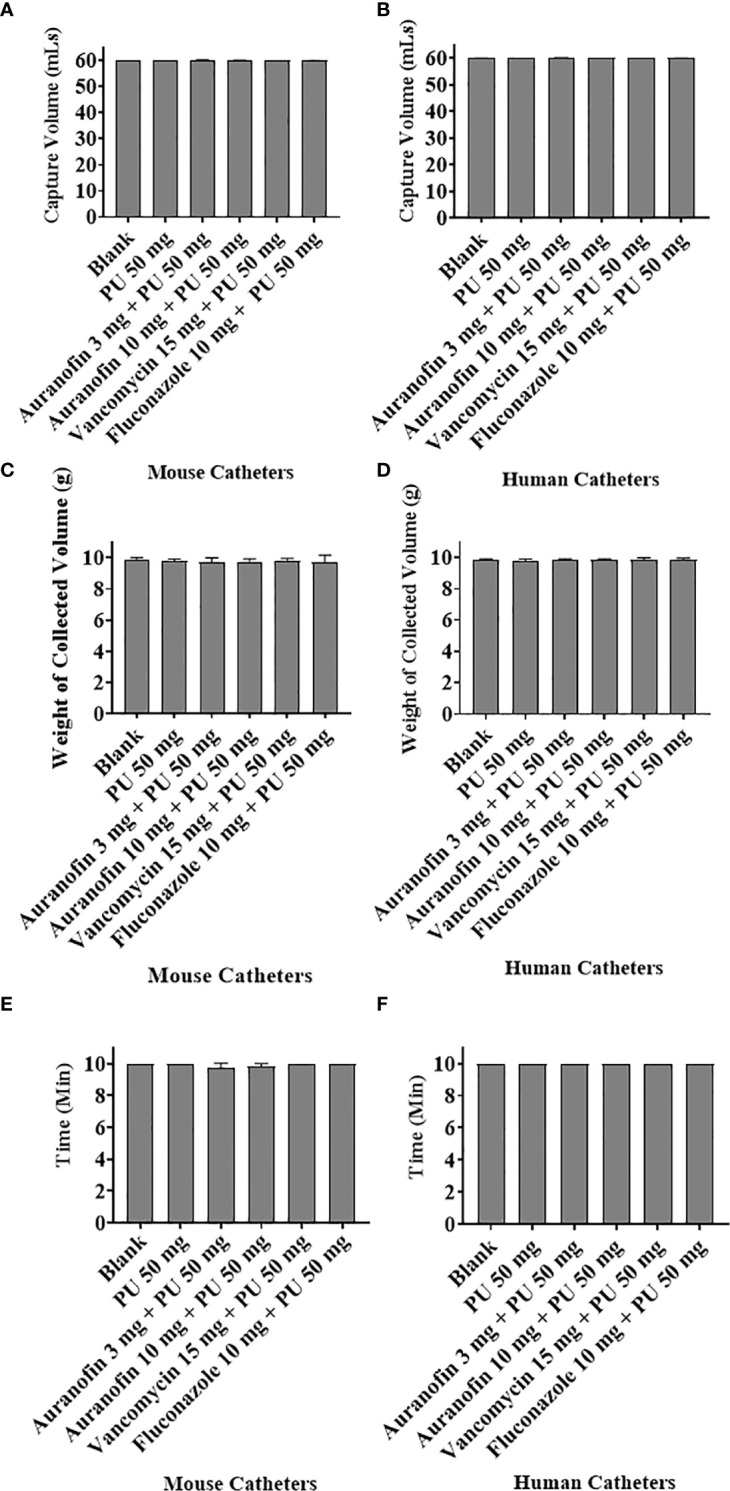
The flow dynamics of drug-coated mouse and human catheters were tested using a Harvard Apparatus syringe pump. **(A, B)** Qualitative analysis evaluated the time for 60 mL of PBS to flow through coated and uncoated mouse and human catheters, keeping the volume constant. **(C, D)** Quantitative analysis weighted total PBS flowing through catheters in 10 min. **(E, F)** The time required for 10 mL of PBS to flow through coated catheters was assessed.

Fluid flow was assessed by passing PBS fluid through catheters at a constant rate of 1 mL/min using a syringe pump **(** **Figure 1** **).** The total collected volume was measured to see if the THF, PU, or drug coatings impeded fluid. The fluid that passed through the dip-coated catheters (mouse and human) remained consistent compared to the uncoated control catheters, achieving a 60 mL collection volume after 1 h as expected with the 1 mL/min flow rate **(** **Figures 1A, B** **)**.

Analytical measurement of the total weight of PBS passing through the catheters at a fixed time of 10 min and at a flow rate set at 1 mL/min found that 9.8 and 9.9 g of PBS passed through the mouse and human catheters, respectively (**Figures 1C, D**) within the designated time frame. Again, indicating that the coating process does not impede fluid compared to the non-coated catheters (no statistical difference compared to the control) and that the total volume flowed through the catheters unimpeded. The average blood flow rate under normal conditions can vary for different parts of the body. The arterial flow rate ranges from 3 to 26 mL/min, and the venous flow rate can be 1.2-4.8 mL/min (Klarhöfer et al., 2001). The tested flow rate of 1 mL/min provides proof of concept. We have provided additional evidence that higher flow rates of 5 mL/min and 10 mL/min do not impact fluid flowing through the coated catheters (5 mL/min and 10 mL/min) **(** **Supplemental Figure 3** **).** The catheters were incubated with 3 mL of PEG400 overnight, and the next day they were removed and dried in a sterile chamber for 24 h. Further, the flow rate of 60 mL PBS was analyzed using a Harvard apparatus. The PEG400 coating was also on human and mouse catheters and used as a comparison with PU coated catheters **(** **Supplemental Figure 4** **)**. There was no significant change in the flow rate in both catheters.

**Figure 2:**
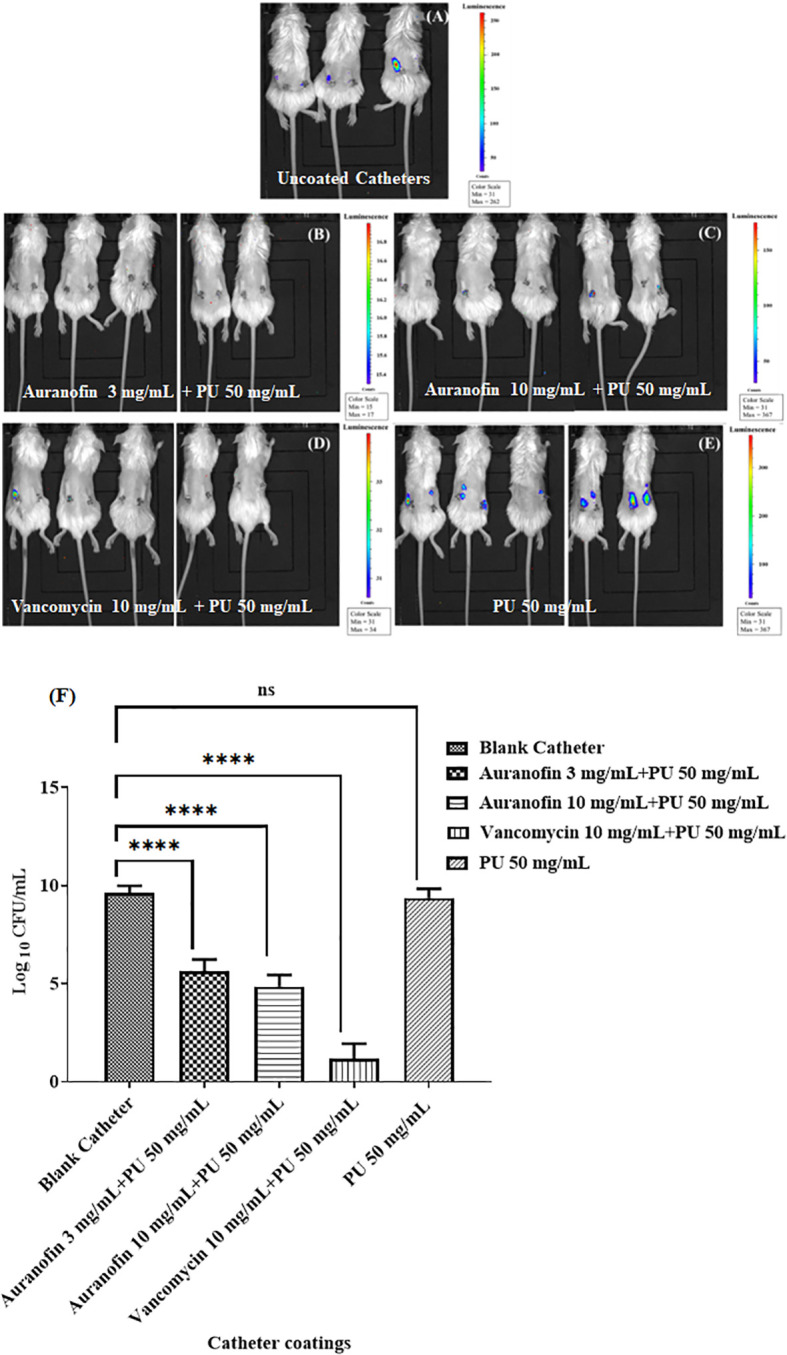
Post implantation, IVIS imaging displayed biofilm accumulation differences in auranofin coated and uncoated catheters in BALB/c mice inoculated with *S. aureus* USA300. **(A)** Uncoated catheter **(B)** Auranofin 3 mg/mL + PU 50 mg/mL **(C)** Auranofin 10 mg/mL + PU 50 mg/mL **(D)** Vancomycin 10 mg/ml + PU 50 mg/mL **(E)** PU 50 mg/mL + THF **(F)** Enumeration of *S. aureus* CFU/mL from recovered catheters. Experiments were performed using 5 animals per group with 2 catheters per animal. Significant differences (*P ≤* 0.05) are shown with an asterisk. Color scale bar indicates *S*. *aureus* density.

### Fluid flow time

The time for a fixed volume of PBS to flow through the catheters was tested to determine if the added coating altered the fluid dynamics. A total volume of 10 mL of PBS was passed through the catheters, with a flow rate set at 1 mL/min, and at optimal flow, the total volume would be collected in 10 min. For both the coated and uncoated catheters, the expected 10 mL volume was recovered in the 10 min period **(** **Figures 1E, F** **)**, indicating that the coating process did not alter the flow rate. Importantly, with the cumulative assessment of volume and time, it appears that neither the carrier PU in THF nor the added antimicrobial drugs altered the flow dynamics compared to uncoated catheters. Although the tested antimicrobial compounds differ in chemistry, size, and hydrophobicity, none adjusted fluid flow based on the tested parameters.

### Auranofin coated catheters inhibit *Staphylococcus aureus* biofilm formation in a murine subcutaneous implant model

Previous *in vitro* investigation of the auranofin and vancomycin-coated catheters demonstrated a significant reduction in *S*. *aureus* biofilm accumulation on catheters, suggesting this specific antimicrobial drug coating impedes bacterial biofilm formation and accumulation on the devices (Liu et al., 2019a). To advance this early finding, coated catheters were implanted subcutaneously, and the bacterial load was measured at the implant site in live animals, visualized *via* luminescence imaging. Captured images showed bacterial accumulation when non-coated catheters were implanted (**Figures 2A-E** **)**. Bacteria were also visualized accumulating at the implant site when catheters were coated with PU dissolved in THF. However, bacterial accumulation was reduced when auranofin or vancomycin was included as part of the catheter coatings.

Post implantation, catheters were recovered from the subcutaneous pockets and *S. aureus* associated with the catheters was enumerated by sonicating the catheter and plating on TSA plates. The uncoated and PU-alone coated catheters accumulated bacteria, reaching 4 x 10^9^ and 1.7 x 10^9^ CFU/mL, respectively. However, a 3 mg/mL auranofin-coated catheter was reduced to 7.4 x 10^5^ CFU/mL, and a 10 mg/mL auranofin-coated catheter reduced bacteria accumulation further to 1.2 x 10^5^ CFU/mL, a 4-log reduction from the controls **(** **Figure 2F** **).** The positive control vancomycin demonstrated the most significant reduction of bacteria, with no bacteria recovered from the catheters. While auranofin efficacy was surpassed by vancomycin (a standard-of-care antibiotic) at the tested concentrations, it provides evidence that auranofin can exert potent bacterial inhibition. Although catheters are coated with mg/mL amounts of compounds, previously published data indicate that µg amounts are released. For example, catheters coated with 10 mg/mL auranofin were found to release approximately 37 µg (Liu et al., 2019a).

### Inhibition of *Candida albicans* biofilm formation on auranofin-coated catheters

**Figure 3:**
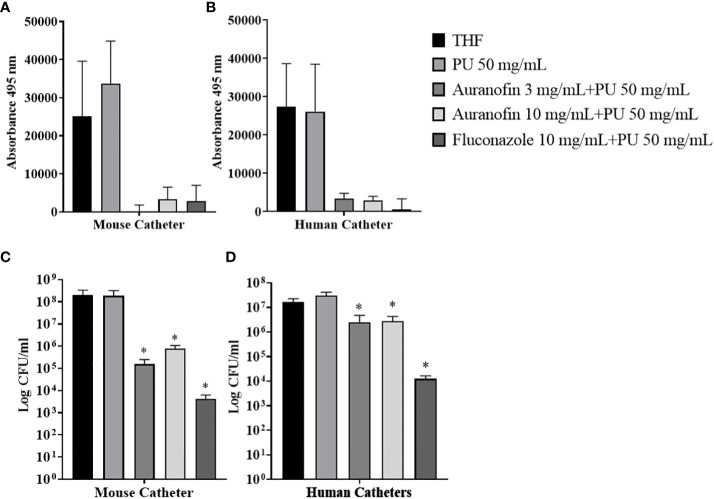
Antimicrobial coatings reduce (*C*) *albicans* biofilm accumulation on catheters. **(A, B)** Quantification of (*C*) *albicans* biofilm formed on catheters coated with 3 mg/mL auranofin, 10 mg/mL auranofin, or 10 mg/mL fluconazole. Biofilms formed on catheters were measured by reading fluorescence from GFP expression. **(B, C)** Catheters were sonicated to dislodge the (*C*) *albicans* biofilm and the material was plated to determine CFU/mL. Significance was determined using Student’s t-test. * Indicates *P*<0.05.

In prior work, we determined that auranofin coated catheters could reduce the accumulation of *S*. *aureus* biofilm *in vitro* (Liu et al., 2019a). The remarkable nature of auranofin is imparted by further testing the impact on fungal accumulation. Indeed, the antimicrobial nature of auranofin has been shown to inhibit both bacterial and fungal pathogens (Fuchs et al., 2016), making it uniquely suited as a catheter coating that can be exposed to numerous types of microbes. A constitutively expressing GFP *C*. *albicans* strain (MLR62) was used to monitor biofilm accumulation on the catheters. The absorbance measurement for *C*. *albicans* GFP expression showed a decrease in biofilm formation on auranofin (3 mg and 10 mg) coated catheters compared to the uncoated catheters **(** **Figures 3A, B** **)**. No GFP was recorded with the 3 mg auranofin-coated mouse catheter, suggesting no detectable accumulation of *C*. *albicans* cells.

Enumeration of *C*. *albicans* cells on auranofin-coated and uncoated catheters (mouse and human) found a reduction attributed to auranofin coating. When coated with 3 mg and 10 mg auranofin, mouse catheters accumulated 1.6 x 10^5^ and 7.8 x 10^5^ CFU compared to 2.0 x 10^8^ CFU associated with catheters exposed to THF solvent alone (**Figure 3C**). Thus, a 3-log reduction in *C*. *albicans* cells was achieved with the auranofin coating with *P*=0.0229 and *P*=0.023, respectively. When the dip coating method was applied to human catheters, associated *C*. *albicans* cells were also reduced from accumulating on the material. Catheters exposed to THF solvent were found to have 1.6 x 10^7^ CFU compared to 2.5 x 10^6^ (*P*=0.0052) and 2.8 x 10^6^ (P=0.0049) CFU recovered from catheters coated with 3 and 10 mg/mL auranofin, respectively **(** **Figure 3D** **)**. Fluconazole-coated catheters (1x10^4^ CFU/mL) also demonstrated a reduction in *C*. *albicans* accumulation. Measuring *C*. *albicans* biofilm biomass can be challenging because this requires weighing the catheters prior to introducing the *C*. *albicans* post-biofilm formation. In this case, there can be variations in the coating volume added to the individual catheters due to slight differences in release volumes during the incubation period that can impact weight. Thus, mass cannot be accurately measured. Therefore, we used *C*. *albicans* strain MLR62 which constitutively expresses GFP as a means of measuring live *C*. *albicans* cells associated with the catheters. To provide insights into the impact of auranofin on *C*. *albicans* biofilm accumulations, *C*. *albicans* biofilm biomass changes on silicone pads were demonstrated in the presence of auranofin **(** **Supplemental Figure 5** **)**. Treating wells with auranofin demonstrate that *C*. *albicans* biofilm accumulation is reduced compared to untreated control wells based on dry weight. Therefore, the result demonstrated that mouse and human catheters coated with auranofin exhibited a significant reduction in *C. albicans* biofilm formation and accumulation over the 60-hour test period, enough time for mature biofilms to form.

### Inhibition of mixed *Staphylococcus aureus* - *Candida albicans* biofilm formation on auranofin-coated catheters

**Figure 4:**
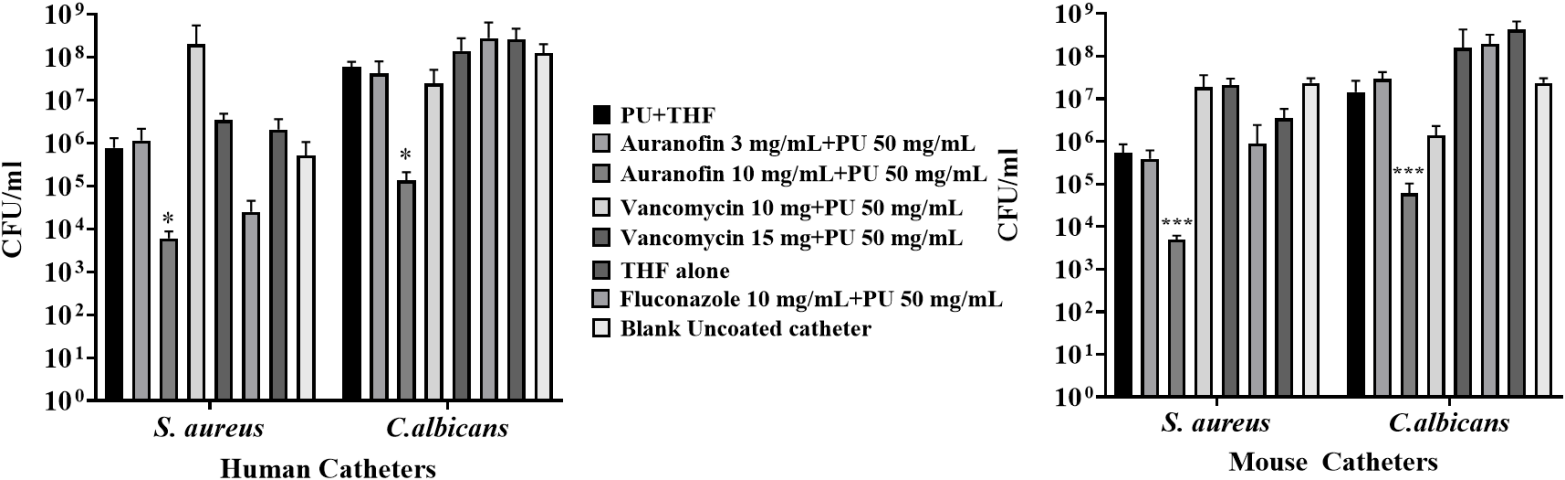
Enumeration of bacterial and fungal biofilm formed on auranofin coated and uncoated mouse and human catheters. Biofilms were allowed to form, and after 60 hours, the catheters were washed and sonicated to collect and enumerate attached cells. Statistical analysis was carried out using Student’s t-test. Significant differences (*P ≤* 0.05) are shown with an asterisk.

A unique feature of auranofin is its ability to inhibit both bacterial and fungal pathogens (Harbut et al., 2015; Fuchs et al., 2016). To evaluate the ability of auranofin to impact a mixed microbe biofilm, catheters were exposed to a mixed pathogen culture of *S. aureus* and *C. albicans* biofilm allowed to mature before being disrupted by sonication and plated on TSA and YPA selection media to enumerate the individual microbes. Applying 10 mg auranofin on human catheters reduced *S*. *aureus* accumulation from 5.1 x10^5^ CFU/mL found on non-coated catheters to 6 x10^3^ CFU/mL but failed to reach significance. However, *C. albicans* biofilm on the human catheter was reduced from 1.2 x 10^8^ CFU/mL on non-coated catheters to 1.3 x 10^5^ CFU/mL on the 10 mg auranofin coated catheters, an efficient reduction (*P*=0.0163) as demonstrated with a Student’s t-test (**Figure 4A**). Although both vancomycin and fluconazole impacted microbe accumulation on human catheters, they failed to have cross-species efficacy as seen with high concentrations of auranofin. Fluconazole reduced *S*. *aureus* accumulation from 5.1 x10^5^ CFU/mL found on non-coated catheters to 2.5 x10^4^ CFU/mL (*P*=0.1262) and did not reduce *C*. *albicans* accumulation (2.8 x 10^8^ CFU/mL; *P*=0.4312). Vancomycin coating at 15 mg caused an increase in *S*. *aureus* associated with the catheters, reaching 3.4 x 10^6^ CFU/mL. *C*. *albicans* accumulation was also not reduced by vancomycin, reaching 2.43 x 10^7^ CFU/mL with the 10 mg coating concentration.

Murine catheters were also evaluated in the same manner. Catheters coated in 10 mg auranofin exhibited *S. aureus* reduction from 2.3 x 10^7^ CFU/mL to 5 x 10^3^ CFU/mL (*P*=0.0005) (**Figure 4B**). *C. albicans* biofilm was reduced from 2.3 x 10^7^ CFU/mL on non-coated catheters to 6 x 10^4^ CFU/mL when coated with 10 mg of auranofin (*P*=0.0005) **(** **Figure 4B** **)**. Thus, both catheters coated with auranofin 10 mg + PU significantly reduced microbe accumulation on the catheters under *in vitro* conditions that usually promote microbial attachment and mature biofilm formation.

### Auranofin coated catheters inhibit *Candida albicans* biofilm formation in a murine subcutaneous implant model

**Figure 5:**
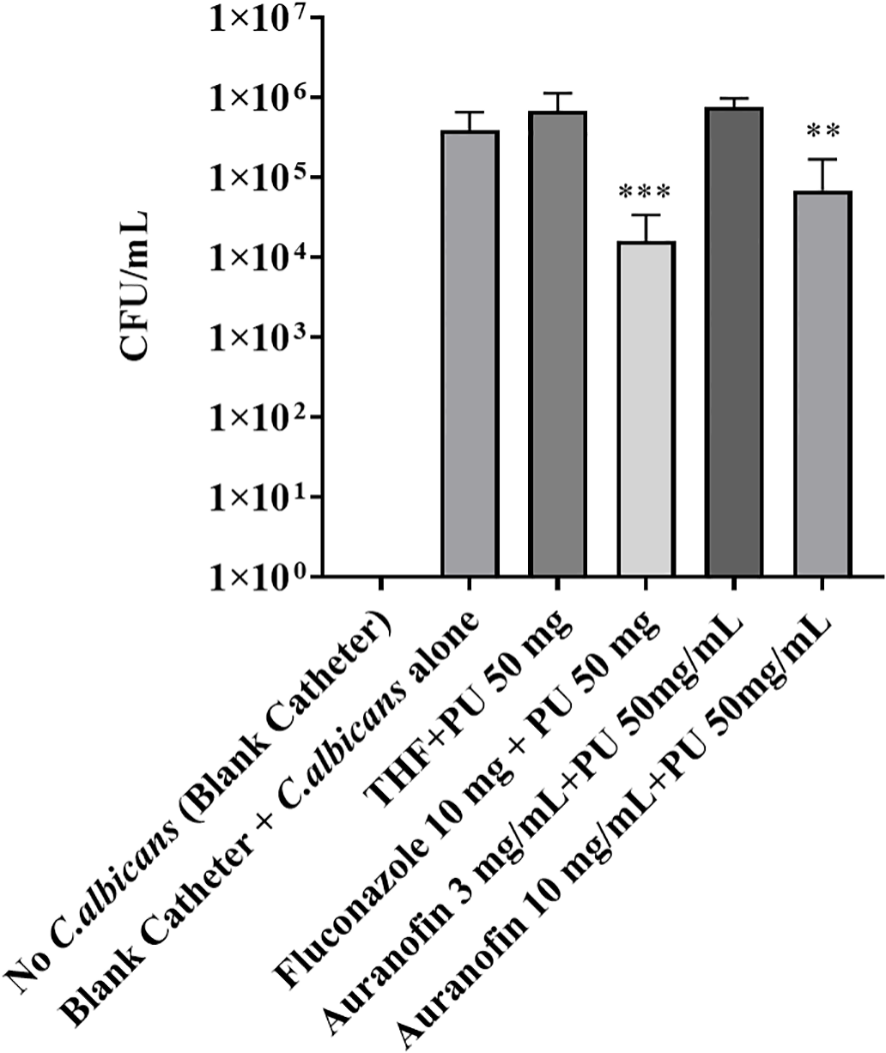
Auranofin coated catheters significantly reduced *C. albicans* biofilm on catheters in a subcutaneous murine model. Catheters were recovered from mice (day 4), and the materials were sonicated to release attached cells. The collected cells were enumerated for *C. albicans* CFU/mL on YPD agar plates. *C. albicans* biofilm reduction showed 6.9 x 10^4^ CFU/mL (*P*=0.0019) on auranofin coated catheters (10 mg/mL). The uncoated (blank) catheters had 3.9 x10^5^ CFU/mL of *C. albicans* biofilm. Statistical analysis was carried out using Student’s t-test. Experiments included 5 animals per group with 2 catheters per animal. Significant differences (*P ≤* 0.05) are shown with an asterisk.

Finding that auranofin-coated catheters demonstrated *in vitro* reduction of *C*. *albicans* biofilm accumulation prompted further evaluation using a subcutaneous murine model. *C. albicans* cells were allowed to attach to coated catheters before implantation. At 4 days post-implantation, catheters were removed, sonicated to detach *C. albicans*, and the CFU was enumerated on YPD plates. Both fluconazole (10 mg/mL) and auranofin-coated catheters (10 mg/mL) exhibited a reduction in the *C. albicans* biofilm, recovering 1.6 x 10^4^ CFU/mL (*P*=0.0003*)* and 6.9 x 10^4^ CFU/mL (*P*=0.0019), respectively, compared to the non-coated catheters (3.9 x10^5^ CFU/mL) (**Figure 5**). Biofilm reductions depict the efficacy of the coating method and material. The study mainly focuses on testing the efficacy of polyurethane coating with auranofin on catheters. Also, increasing the concentration of auranofin will significantly decrease the biofilm at higher folds in comparison to uncoated catheters. We noted that there was no significant inflammation on the site of catheter insertion in the mouse model. We also collected blood by heart puncture, but no microbe growth was recorded.

## Discussion

Infections involving implant devices impact patient health, recovery time, and overall mortality rates. Prevention practices are essential and reduce widespread nosocomial infection, but more elements can be addressed to improve patient outcomes. Coating catheters have been a means to prevent bacterial attachment and biofilm formation (Suresh et al., 2019). While numerous drugs can be incorporated into the coating process, auranofin presents a particularly intriguing option as an agent that impacts both *S. aureus* and *C. albicans*, representing efficacy against Gram-positive bacteria and fungi (Fuchs et al., 2016; Liu et al., 2019a).

*C. albicans* and *S. aureus* are responsible for most opportunistic nosocomial infections (Harriott and Noverr, 2011), and can be co-isolated from the same host (Krüger et al., 2019). It is common for these polymicrobial infections to be associated with mixed biofilms formed in catheters or other indwelling devices, where *C. albicans* and *S. aureus* display symbiotic relationships (Peters et al., 2010; Todd and Peters, 2019). The use of antimicrobial-coated catheters has been suggested to decrease the risk of acquiring a catheter-related bloodstream infection, and biofilm-inhibiting antimicrobial compounds have been proposed to prevent colonization and biofilm formation (Wang et al., 2018; Zhu et al., 2019).

Auranofin has demonstrated antimicrobial efficacy against bacteria and fungi (Siles et al., 2013; Harbut et al., 2015; Fuchs et al., 2016), suggesting that it could inhibit both types of pathogens which cause catheter-related infections. Further, auranofin impacts bacterial and fungal planktonic cells and biofilms (Siles et al., 2013; Torres et al., 2016; She et al., 2020). In their study, Siles and colleagues demonstrated that auranofin caused 94% *C. albicans* biofilm inhibition (Siles et al., 2013). In our previous study, we used auranofin coating on intravenous catheters to prevent the biofilm of *S. aureus* under *in vitro* conditions (Liu et al., 2019a). As a continuation of the previous work, this report shows the efficacy of auranofin-coated catheters against *S. aureus* and *C. albicans* biofilm under *in vitro* and *in vivo* conditions. This is the first report of auranofin-coated catheters inhibiting mixed biofilm of *S. aureus*- *C. albicans*.

Despite coating mouse and human catheters with auranofin, there was no change in the flow, volume, and weight of liquid passing through the coated catheter. This suggested that PU and auranofin coating on the catheter does not influence the flow rate concerning time or volume. But in a study by (Gefter Shenderovich et al., 2018), chlorhexidine coated using polyethylene glycol on a catheter affected the release rate due to thicker coating and less stability. Thus, the present study demonstrates PU as an efficient coating material with better flow dynamics.

Previous reports suggested that coating catheters with silver nanoparticles (Lara and Lopez-Ribot, 2020) and antibiotics (Vila et al., 2021) provided efficient inhibition of mixed biofilm of *S. aureus* and *C. albicans*. Antimicrobial catheters should offer long-term antimicrobial surface effects without side effects or toxicity and a low application cost (Singha et al., 2017). In the present study, catheter coated with PU + auranofin offers an attractive alternative to prevent the colonization and biofilm formation of *S. aureus* and *C. albicans* while not taxing current standard of care antibiotic or antifungal therapeutic agents, thus respecting antimicrobial stewardship. The auranofin-coated catheter showed efficient anti-biofilm activity against the mixed microbe biofilm composed of *S. aureus* and *C. albicans*. In addition to auranofin, both the control antimicrobial agents (fluconazole and vancomycin) were able to act as effective coating agents against *C. albicans* and *S. aureus*, but the standard of care controls failed to inhibit microbial accumulation from both *S*. *aureus* and *C*. *albicans* showing the inability to affect dual biofilms. The difference in biofilm formation of *C. albicans* on the mouse and human catheter is due to the different materials used for manufacturing them. The mouse catheters are made with polyurethane, while human catheters are made using radiopaque teflon.

In conclusion, *in vitro* assays demonstrated that auranofin-coated catheters inhibit *S. aureus* and *C. albicans* biofilm formation. Qualitative and quantitative analysis of auranofin-coated catheter in a murine subcutaneous implant model showed reduced biofilm formation. Both *S. aureus* and *C. albicans* subcutaneous murine models demonstrated that catheters coated with 10 mg of auranofin experienced reduced microbe accumulation. Although vancomycin is an effective antimicrobial that inhibits *S*. *aureus* planktonic cells, it is not as impactful against biofilm. Further, although coated at 10 mg/mL, a previous study by (Liu et al., 2019a) demonstrated that coating concentration greatly exceeded release concentration (Liu et al., 2019a) and in this case, it’s likely much vancomycin release concentration is much lower. Thus, it fails to be impactful in reducing *S*. *aureus* biofilm accumulation on catheters. Successful reduction in biofilm formation indicated that auranofin is an excellent candidate for catheter coating against bacterial and fungal biofilm without altering catheter flow dynamics. The cumulative results indicate that the dip-coating method successfully provides a protective coating on the catheters and *S*. *aureus* and *C*. *albicans* biofilm formation is reduced by auranofin application.

## Data availability statement

The original contributions presented in the study are included in the article/**Supplementary material**. Further inquiries can be directed to the corresponding author.

## Ethics statement

The animal study was reviewed and approved by Institutional Animal Care and Use Committee.

## Author contributions

LF and BF designed and carried out experiments. CW, NV-G, and AS helped with the coating of catheters. NT and BM assisted with animal experiments. BF, EM, and AS directed research, provided insightful discussion, and contributed to the writing. All authors contributed to the article and approved the submitted version.

## Supplementary material

Supplementary Figure 1 Schematic of flow rate assessment for coated and uncoated mouse and human catheters. 1. Syringe pump (Harvard apparatus). 2. Fluid-filled syringe. 3. Connecting syringe adapter. 4. Catheter. 5. Collection tube.

Supplementary Figure 2
*In vivo* assessment of *S*. *aureus* biofilm accumulation. Catheters incubated with *S*. *aureus* USA300 were implanted into subcutaneous pockets on either side of the mouse flanks. Post incubation, the catheters were recovered and sonicated to dislodge attached cells, and the slurry was plated on selection media to enumerate the CFU on each catheter.

Supplementary Figure 3 The flow kinetic of the drug-coated mouse and the human catheter was tested using a Harvard Apparatus syringe pump. The analysis evaluated the time for 60 mLs of PBS to flow through coated and uncoated mouse and human catheters, keeping the volume constant at 5 mL/min and 10 mL/min.

Supplementary Figure 4 PEG400 coating was applied to the mouse and human catheters to determine the flow rate of 60 mL PBS. The flow rate was fixed at 1 mL/min for a total time of 60 min. No significant changes were observed in the flow rate compared to PU-coated catheters.

Supplementary Figure 5 The different concentration of auranofin and fluconazole was tested on *C. albicans* MLR62 biofilm. The *C. albicans* biofilm was formed on a silicone pad in the presence of auranofin and fluconazole, and the biomass was weighed after the incubation period. The experiments were performed in triplicate.
