# Supplementary figures and images for: Auranofin coated catheters inhibit bacterial and fungal biofilms in a murine subcutaneous model
Source: Front Cell Infect Microbiol. 2023 May 29;13:1135942. doi: 10.3389/fcimb.2023.1135942 (PMC10258325; doi:10.3389/fcimb.2023.1135942)

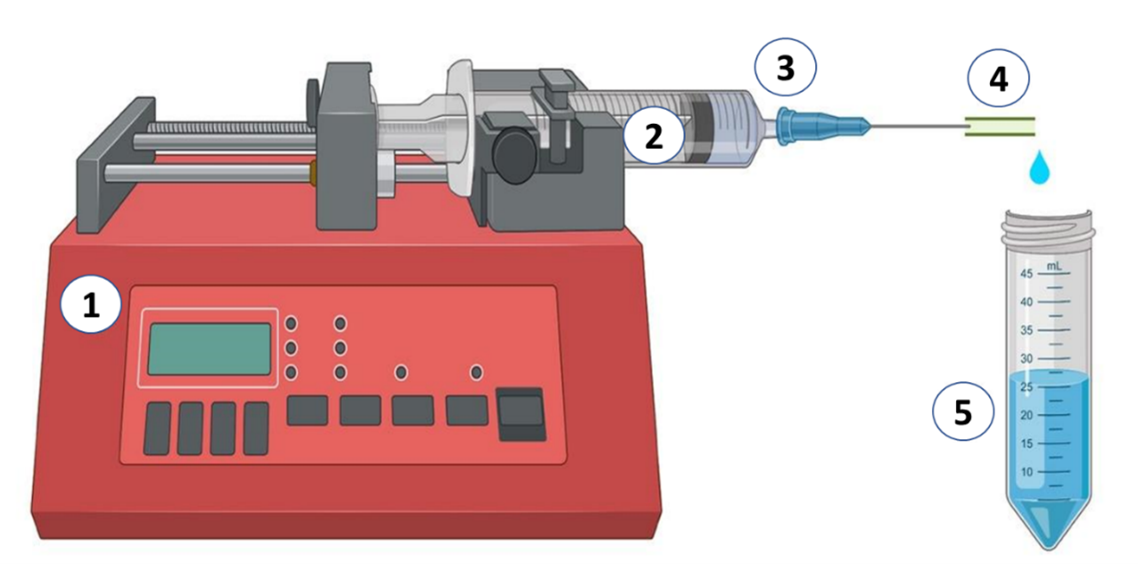

Supplement: Supplementary Figure 1 — Schematic of flow rate assessment for coated and uncoated mouse and human catheters. 1. Syringe pump (Harvard apparatus). 2. Fluid-filled syringe. 3. Connecting syringe adapter. 4. Catheter. 5. Collection tube. [file Image_1.tif]

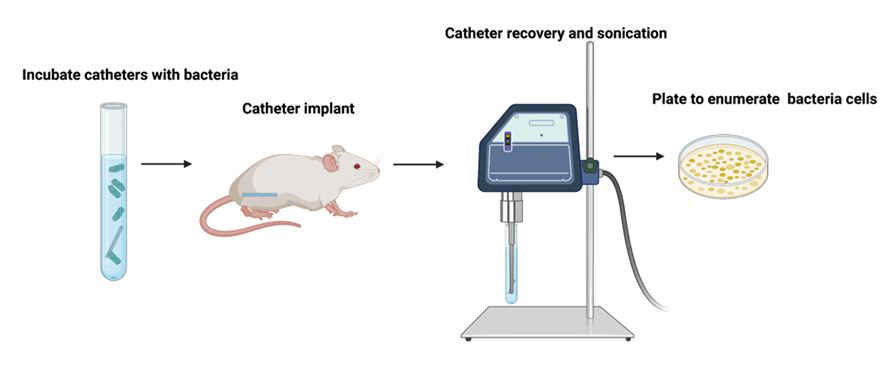

Supplement: Supplementary Figure 2 — In vivo assessment of S. aureus biofilm accumulation. Catheters incubated with S. aureus USA300 were implanted into subcutaneous pockets on either side of the mouse flanks. Post incubation, the catheters were recovered and sonicated to dislodge attached cells, and the slurry was plated on selection media to enumerate the CFU on each catheter. [file Image_2.tif]

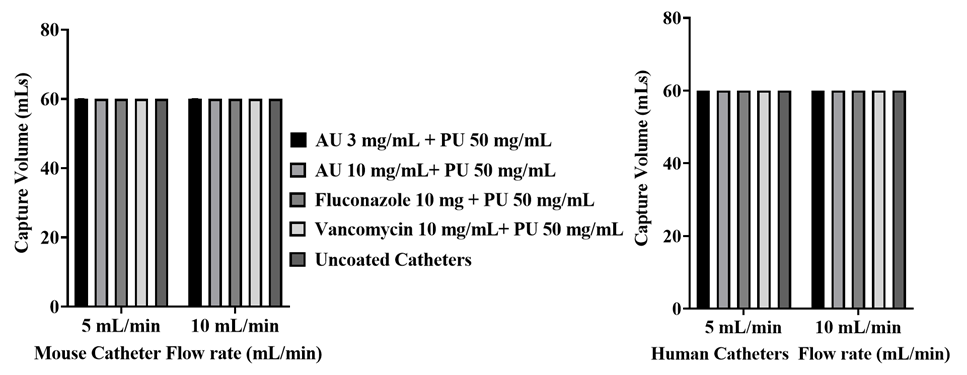

Supplement: Supplementary Figure 3 — The flow kinetic of the drug-coated mouse and the human catheter was tested using a Harvard Apparatus syringe pump. The analysis evaluated the time for 60 mLs of PBS to flow through coated and uncoated mouse and human catheters, keeping the volume constant at 5 mL/min and 10 mL/min. [file Image_3.tif]

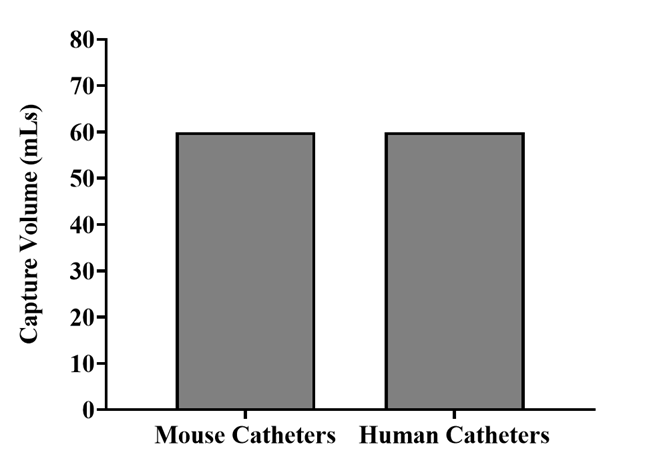

Supplement: Supplementary Figure 4 — PEG400 coating was applied to the mouse and human catheters to determine the flow rate of 60 mL PBS. The flow rate was fixed at 1 mL/min for a total time of 60 min. No significant changes were observed in the flow rate compared to PU-coated catheters. [file Image_4.tif]

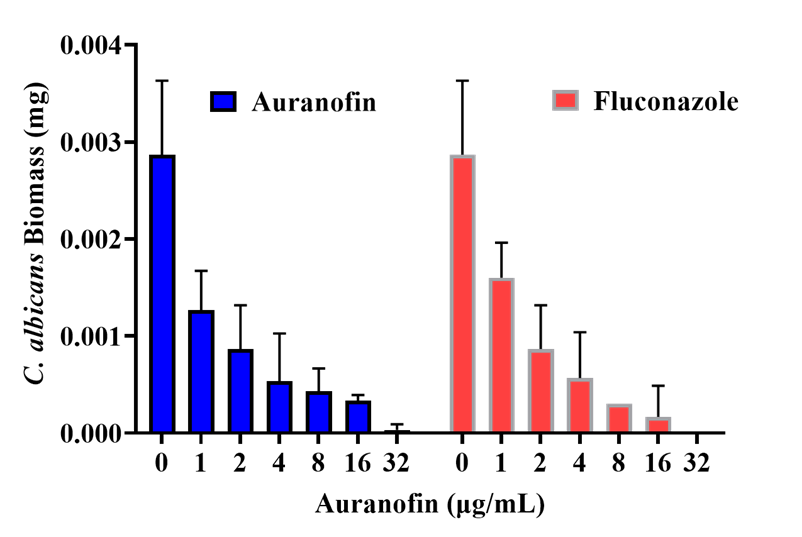

Supplement: Supplementary Figure 5 — The different concentration of auranofin and fluconazole was tested on C. albicans MLR62 biofilm. The C. albicans biofilm was formed on a silicone pad in the presence of auranofin and fluconazole, and the biomass was weighed after the incubation period. The experiments were performed in triplicate. [file Image_5.tif]
