# Supplementary material for: Auranofin coated catheters inhibit bacterial and fungal biofilms in a murine subcutaneous model
Source: Front Cell Infect Microbiol. 2023 May 29;13:1135942. doi: 10.3389/fcimb.2023.1135942 (PMC10258325; doi:10.3389/fcimb.2023.1135942)
Supplement: Supplementary file 6 [file DataSheet_1.docx]

**Supplementary Materials and Methods**

**Coating of mouse and human catheters with PEG400**

The mouse and human catheters were coated with auranofin cut into 1 cm segments using a sharp scalpel, and submerged in a 20 mL scintillation vial containing 3 mL of PEG-400. The setup was placed on a shaker overnight at room temperature. Post incubation, catheters were air-dried in a fume hood overnight and then stored at 4 ºC until further use.

**Fluid flow dynamics with coated catheters**

For both human and mouse catheters, assessment groups included: untreated catheters, catheters coated with auranofin (3 and 10 mg) suspended in THF + PU coating, vancomycin (15 mg) suspended in THF + PU coating, and fluconazole (10 mg) suspended in THF + PU coating. The flow of PBS through human and mouse catheters was determined by keeping the volume of liquid constant. A 30 mL syringe was filled with 30 mL (the syringe was filled 2 times) of PBS and connected to a catheter adapter that married the catheters to the syringe tip, connecting the devices to the syringe pump, which was then set to run at a flow rate 5 mL/min and 10 mL/min at a 50% force level to assess the final volume captured during a fixed period.

**Inhibition of *C. albicans* biofilm accumulation on a silicone pad using auranofin**

Silicone pads were cut in 1x1 cm squares and sterilized. The sterile silicone pads were weighed and treated with 2 ml of Bovine Serum (ThermoFisher Scientific, Waltham, MA) overnight at 37 °C with shaking in a 12-well plate (150 rpm). After treatment, bovine serum was removed, and silicone pads were washed with 2 mL of PBS and then placed in a 12-well plate with 2 mL of Spider media. An overnight culture of C. albicans MLR62 in YPD at (OD_600_ = 0.5) was added to the 12-well plate containing a silicone pad along with designated concentrations of auranofin or fluconazole. The untreated silicone pad with C. albicans alone was used as a control and silicone pads without *C*. *albicans* inoculation were used as microbial growth control. Plates were incubated for 90 min at 37 °C with agitation (150 rpm). After incubation, the silicone pads were washed with PBS, and to this, again the designated concentrations (1, 2, 4, 8, 16, and 32 µg/mL) of auranofin and fluconazole were added. The silicone pads were incubated for 60 h at 37 °C. After incubation, the silicone pad was gently washed with 2 mL PBS and dried overnight in the hood. The silicone pads were weighed again to determine the weight of the biofilm formed on the silicone pad. The pre and post biofilms formation weights of the silicone pad were subtracted to determine the biofilm mass formed on the silicone pad.
